# Evolution of Digital Health and Exploration of Patented Technologies (2017-2021): Bibliometric Analysis

**DOI:** 10.2196/48259

**Published:** 2024-07-11

**Authors:** Wenjun Gu, Jinhua Wang, Yunqi Zhang, Shaolin Liang, Zisheng Ai, Jiyu Li

**Affiliations:** 1 School of Medicine Tongji University Shanghai China; 2 Shanghai Institute of Medical Innovation Technology Transfer Shanghai China; 3 STI-Zhilian Research Institute for Innovation and Digital Health Beijing China; 4 Institute for Six-sector Economy Fudan University Shanghai China; 5 Geriatric Oncology Center Huadong Hospital Affiliated to Fudan University Shanghai China

**Keywords:** technology trends, digital health, patent, bibliometric analysis, CiteSpace5.1R8

## Abstract

**Background:**

The significant impact of digital health emerged prominently during the COVID-19 pandemic. Despite this, there is a paucity of bibliometric analyses focusing on technologies within the field of digital health patents. Patents offer a wealth of insights into technologies, commercial prospects, and competitive landscapes, often undisclosed in other publications. Given the rapid evolution of the digital health industry, safeguarding algorithms, software, and advanced surgical devices through patent systems is imperative. The patent system simultaneously acts as a valuable repository of technological knowledge, accessible to researchers. This accessibility facilitates the enhancement of existing technologies and the advancement of medical equipment, ultimately contributing to public health improvement and meeting public demands.

**Objective:**

The primary objective of this study is to gain a more profound understanding of technology hotspots and development trends within the field of digital health.

**Methods:**

Using a bibliometric analysis methodology, we assessed the global technological output reflected in patents on digital health published between 2017 and 2021. Using Citespace5.1R8 and Excel 2016, we conducted bibliometric visualization and comparative analyses of key metrics, including national contributions, institutional affiliations, inventor profiles, and technology topics.

**Results:**

A total of 15,763 digital health patents were identified as published between 2017 and 2021. The China National Intellectual Property Administration secured the top position with 7253 published patents, whereas Koninklijke Philips emerged as the leading institution with 329 patents. Notably, Assaf Govari emerged as the most prolific inventor. Technology hot spots encompassed categories such as “Medical Equipment and Information Systems,” “Image Analysis,” and “Electrical Diagnosis,” classified by Derwent Manual Code. A patent related to the technique of receiving and transmitting data through microchips garnered the highest citation, attributed to the patentee Covidien LP.

**Conclusions:**

The trajectory of digital health patents has been growing since 2017, primarily propelled by China, the United States, and Japan. Applications in health interventions and enhancements in surgical devices represent the predominant scenarios for digital health technology. Algorithms emerged as the pivotal technologies protected by patents, whereas techniques related to data transfer, storage, and exchange in the digital health domain are anticipated to be focal points in forthcoming basic research.

## Introduction

### Background

Over the past few decades, the intertwining of health care and digital technology has given rise to significant transformations in the production, alignment, and consumption of health care products [1]. This integration has contributed to the achievement of safer and more cost-effective health care outcomes. The conceptual emergence of terms such as “Digital Health,” “Digital Medicine,” and “Digital Therapeutics” is a direct consequence of this symbiotic relationship. The International Digital Therapeutics Alliance (DTA), founded in 2017 in the United States, stands as a nonprofit industry association comprising stakeholders committed to evidence-based therapeutic interventions aimed at preventing, managing, or treating diseases [2]. As per the DTA’s classification, digital therapeutics represents a specific niche within digital health and digital medicine [3]. Notably, a range of digital therapeutics products is currently available for managing diabetes [4,5], treating patients with social anxiety disorder [6] or neurological disorders [7], addressing mental illness [8], and developing digital biomarkers designed to predict treatment response [9]. These products rely on real-world data, and clinical evidence is imperative to substantiate claims regarding risk, efficacy, and intended use [10].

Nielsen and Sahay [11] conducted a critical examination aimed at identifying gaps in the literature, approaching the analysis from an information systems perspective. Their study, based on the analysis of 342 articles published in interdisciplinary digital health research journals, revealed that these studies tended to deviate from the complexities inherent in real-life settings within health care organizations, particularly in relation to the characteristics of digital technologies and the context of their use. Notably, the literature on digital health primarily emphasized the processing power of digital technology and its potential effects [11]. In line with the observations from the study by Nielsen and Sahay [11], a bibliometric analysis conducted by Keng Yang et al [12] in 2022 spanning the period from 1998 to 2021 demonstrated a rapid expansion in the number of publications on digital health. The findings suggested that heightened awareness of digital health had the potential to improve health outcomes, bridge digital gaps, and reduce health disparities [12]. Supporting this perspective, Ahmadvand et al [13] used “digital health” as a keyword for their bibliometric analysis, covering articles published between January 2000 and August 2019 in JMIR Publications journals. Their study revealed a significant increase in the number of articles focusing on “digital health” over a 9-year period, with “mHealth” emerging as the most frequently used keyword within this domain [13]. In addition, Gupta et al [14] conducted a scientometric assessment of digital health research, examining 6981 global publications sourced from the Scopus database during the period 2007 to 2016. Their research underscored medicine as the dominant topic, constituting the largest publication share in digital health research at 54% [14].

Previous studies primarily focused on published papers, neglecting the distinctive value provided by patents as a special and unique source of knowledge, given that a significant amount of data and information contained in patents is not made available through alternative channels. The patent system, designed to stimulate innovation, concurrently ensures that the benefits of inventive efforts are made accessible to the public. In the field of digital health, considering the industry’s rapid and iterative development, the patent system must leverage digital technology to safeguard algorithms, software, and advanced surgical instruments. Apart from furnishing information on digital health patents, the patent system serves an additional role by granting other researchers access to patent information, thereby facilitating enhancements in existing technologies.

Bibliometric analysis serves as a powerful tool for examining research trends, identifying prolific authors, understanding demographics, and exploring related information within specific fields [15]. CiteSpace5.1R8, a widely used scientific mapping software application, is adept at analyzing hot spots and trends, visually illustrating a systematic understanding of the past across various domains [16]. Bibliometrics, along with visual analysis, of patent landscapes in the fields of digital technology within public health is relatively scarce. Therefore, this study predominantly relies on this methodology to investigate granted patents related to digital technology in health care, shedding light on its technical status and prevailing tendencies.

### Objectives

The research uses the bibliometric method to delve into the annual volume of patent applications and grants, scrutinizing information related to countries, inventors, patentees, cited patents, and Derwent Manual Code (DMC) classifications of digital technologies. The primary objective of this study is to gain a more profound understanding of technology hot spots and development trends within the field of digital health.

## Methods

### Data Collection

**Figure 1:**
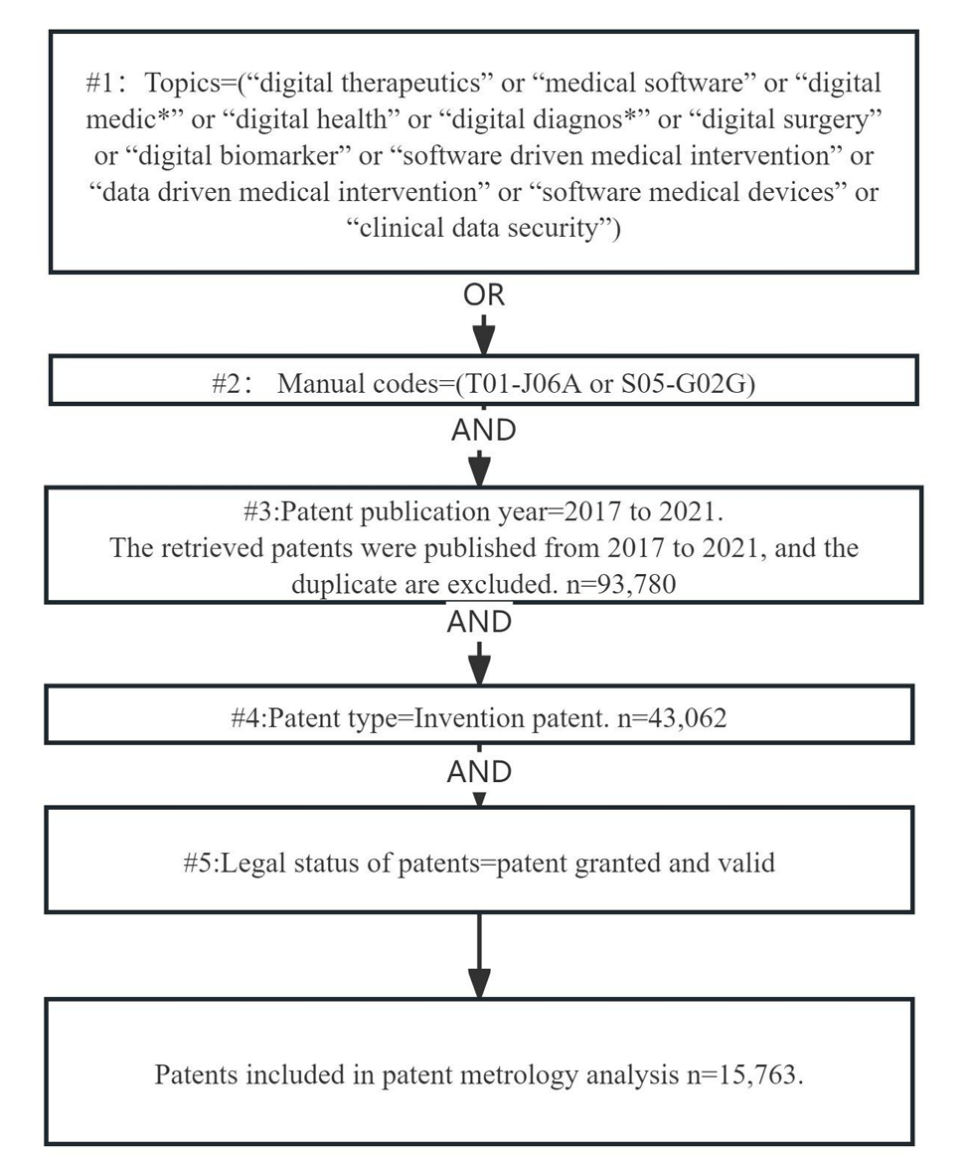
The flowchart for data collection.

The data for this study were sourced from the Derwent Innovation Index, a database that amalgamates patent citations from the Derwent Patents Citation Index with additional patent data indexed from >50 patent issuing agencies in the Derwent World Patent Index (1963 to present). Adhering to the procedural guidelines by Chen [17], we retrieved patents associated with “digital health,” “digital medicine,” and “digital therapeutics,” along with a selection of other pertinent topics. Our screening strategies incorporated DMC “T01-J06A,” signifying “medical equipment and information systems,” and “S05-G02G,” representing “hospital equipment with medical IT systems,” based on the categorization of DMC. DMC serves as a simplified classification system designed to categorize patent documents across all technologies. This coding system streamlines the categorization and indexing method used by Derwent for all covered patents, ensuring efficiency [18,19]. Figure 1 illustrates our specific screening strategies in the flowchart for data collection.

The patent retrieval process involved several steps to compile a comprehensive data set related to digital health. Initially, we extracted patents with topics relevant to digital health using the topic tag for string retrievals through titles and abstracts of patents in the Derwent Innovation Index. The first step (#1) used the following query: Topics=(“digital therapeutics” OR “medical software” OR “digital medic*” OR “digital health” OR “digital diagnosis*” OR “digital surgery” OR “digital biomarker” OR “software-driven medical intervention” OR “data-driven medical intervention” OR “software medical devices” OR “clinical data security”). Subsequently, we retrieved patents using DMC. The query command for the second step (#2) was Manual Codes=(T01-J06A OR S05-G02G). In the third step, we combined the commands from #1 OR #2 and limited the publication date of patents from 2017 to 2021. This timeframe was chosen because significant developments in digital health occurred in 2017, including the establishment of the International DTA, release of guidelines by the Chinese State Food and Drug Administration, publication of the World Health Organization recommendations on digital technologies for Tuberculosis care, and the US Food and Drug Administration approval of the first prescription digital therapy. We retrieved 93,780 published patents using this criterion. Subsequently, in the fourth step, we focused on invention patents, as they generally hold greater value in most countries, resulting in 43,062 invention patents included in the sample. As expired patents lack legal force, our analysis primarily considered patents in force to reflect the latest trends in digital technology in public health. We included patents from the previous step (#4) with a granted and valid legal status, resulting in a final data set of 15,763 patents included in our study sample. According to our search methods, patents from the African region have not been retrieved in this study.

As outlined in the introduction to the Derwent Innovation Index database, the earliest time stamp for data collection extended back to 1963. The initiation of patent retrieval for published patents began in 2017. Given that some patents published in 2017 might have been granted in 2022, the conclusion of the study period was set at the year 2022. The data retrieval process was completed on September 22, 2022.

### Analysis Methodologies

In conducting this study, CiteSpace 5.1 R8 SE and Excel 2016 software were used. By configuring parameters such as time slicing and thresholds, diverse results could be obtained [20,21]. Furthermore, CiteSpace offered the capability to visualize the outcomes of the analysis [22], using linkages and nodes to illustrate the quality of various elements and their interrelationships [23]. The software also had the ability to cluster items using statistical techniques such as log-likelihood ratio and assess the burst of themes to identify DMCs with prolonged bursts and high strength [24]. CiteSpace contributes to enhanced clarity and interpretability of visualizations compared to other tools, thereby reducing cognitive load on users as they explore significant trends and turning points in a technological framework [25,26].

The chosen records were exported from the Derwent Innovation Index in plain text format and subsequently imported into CiteSpace for comprehensive analysis and visualization of cited patents, research hot spots, and frontiers. A total of 15,763 patent records, encompassed by the CiteSpace version, were retrieved based on their patent publishing timeframe spanning from 2017 to 2022. The data were segmented into 1-year time slices, and the top 50 most cited strings were extracted from each slice. The thematic analysis incorporated various fields from the plain texts, encompassing inventors, cited patents, patent assignees, cited authors, DMC, and International Patent Classification (IPC). Node types were specified as country, institution, author, and category, and the results were visualized in this specified order. This approach allowed for a detailed exploration of relationships and patterns within the data set, providing insights into the geographical, institutional, and authorship aspects of digital health patents.

The chosen records were exported from the Derwent Innovation Index in a table format and subsequently imported into Excel 2016 for thorough analysis. The study focused on examining the annual volume of applications, granted patents, the most productive countries, institutions, inventors, highly cited patents, and institutes. This approach in Excel allowed for a systematic exploration of quantitative aspects related to digital health patents, providing insights into the dynamics of patent activity over time and identifying key contributors and influential patents in the field.

## Results

### Applications and Granted Trends Across Diverse Sectors

**Figure 2:**
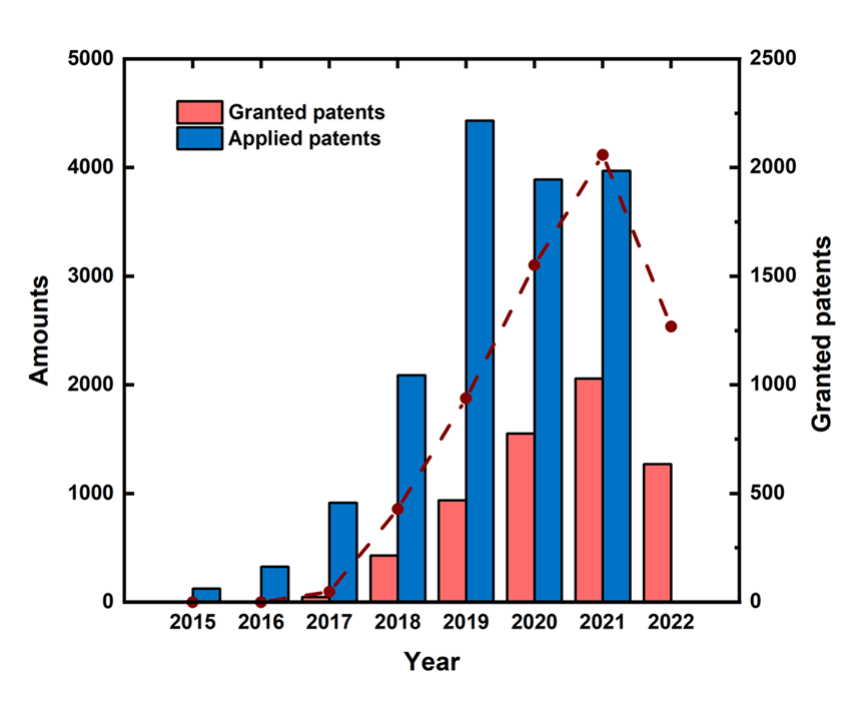
Applied and granted patents from 2017 to 2022.

We conducted an analysis of all 15,763 granted patents related to digital health published between 2017 and 2021. In 2017, a total of 48 patents were granted, followed by a surge to 428 granted patents in 2018. As illustrated in Figure 2, the annual applications for patents in the digital health domain experienced a sharp increase from 2017 to 2019, followed by a slight stagnation in 2020. However, a renewed upward trend was observed from 2020 to 2021. A general upward trajectory was noted in granted patents from 2017 to 2022. The fluctuation in 2022 may be attributed to the timing of data retrieval, as patents published between 2017 and 2021 might not have been granted by September 22, 2022.

### Geographical Distribution

**Figure 3:**
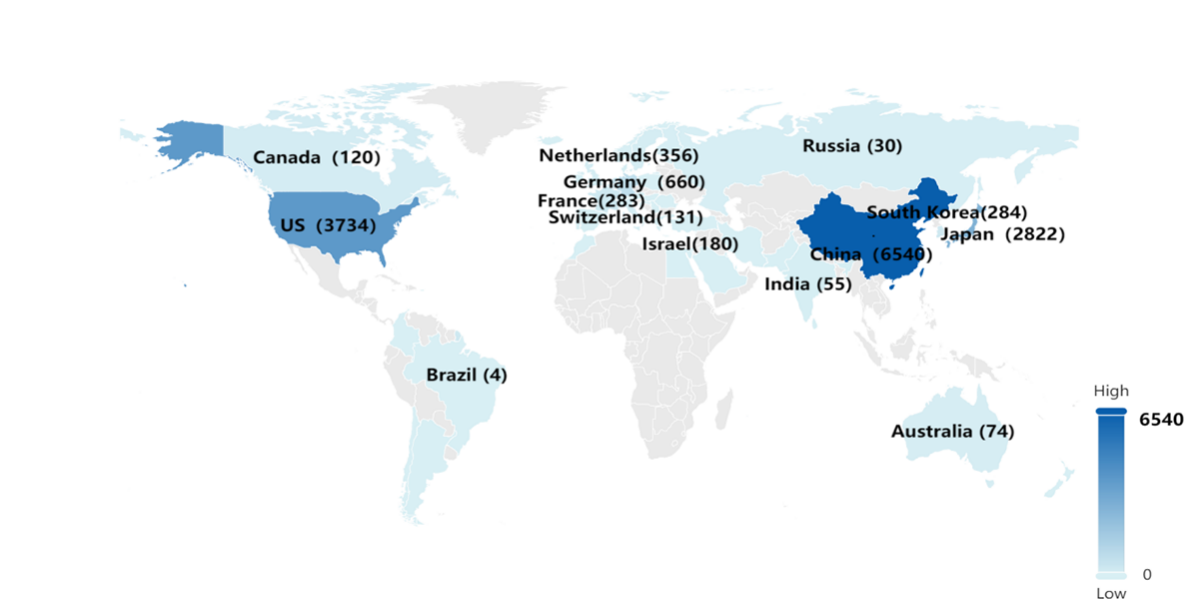
Intellectual property office for published patents (marked according to the country where the intellectual property office is located).

Applicants have the option to apply for patents through intellectual property offices (IPOs) in various countries or regions, thereby extending the scope of protection for their technological innovations in the field of digital health. Table 1 and Figure 3 present the top 10 IPOs for published patents and the top 10 patent applicant countries concerning patent publications. These insights offer a comprehensive view of the global landscape in terms of patent activities related to digital health, highlighting the key contributors and regions involved in the innovation and protection of digital health technologies.

**Table 1:** Top 10 intellectual property offices for published patents (N=15,763).

Intellectual property office	Published patents, n (%)
China National Intellectual Property Administration	^7253 (46.01)^
United States Patent and Trademark Office	4052 (25.71)
Japanese patent office	2174 (13.79)
European patent office	1242 (7.88)
IP Australia	244 (1.55)
Inspirations Property Institution	228 (1.45)
World Intellectual Property Organization	216 (1.37)
Deutsches Patent- und Markenamt	172 (1.09)
Canadian Intellectual Property Office	94 (0.6)
Spanish Patent and Trademark Office	46 (0.29)

A total of 99.73% (15,721/15,763) of the published patents originated from the top 10 IPOs, as detailed in Table 1. The China National Intellectual Property Administration (CNIPA) held the majority share, contributing 46% (n=15,763) published patents, surpassing other IPOs. The United States Patent and Trademark Office (USPTO) secured the second position with 25.71% (4052/15,763), followed by the Japanese patent office (JPO) at 13.79% (2174/15,763), the European Patent Office at 7.88% (1242/15,763), IP Australia at 1.55% (244/15,763), Inspirations Property Institution at 1.45% (228/15,763), and the World Intellectual Property Organization at 1.37% (216/15,763).

A total of 95.86% (15,110/15,763) of the published patents originated from the top 10 applicant countries, as illustrated in Figure 3. Among these, China emerged as the leading country, submitting the highest number of patents at 6540 (41.5%) of the total 15,763 publications. The United States secured the second position, contributing 23.69% (3734/15,763) of the publications, followed by Japan with 17.9% (2822/15,763).

The alignment of the top 3 IPOs with the leading patent applicant countries, namely, China, the United States, and Japan, underscores these regions as highly competitive in safeguarding technological innovations within the realm of digital health. This correlation suggests that institutions, enterprises, and individuals from these countries are at the forefront of technological innovation in the field of digital health. China, the United States, and Japan collectively stand out as leaders in driving advancements and securing intellectual property protection in the dynamic landscape of digital health technologies.

Multimedia Appendix 1 provides an overview of the nationality distribution of patent applicants within the top 3 IPOs. Specifically, 92.72% (2001/2158) of patent applicants to the JPO were from Japan, with 4.45% (96/2158) and 0.65% (14/2158) of applicants originating from the United States and South Korea, respectively. For the CNIPA, 87.08% (6316/7253) of patent applicants were from China, whereas 3.38% (245/7253) and 3.25% (236/7253) were from Japan and the United States, respectively. In the case of the USPTO, 69.32% (2953/4260) of patent applicants were from the United States, with 6.1% (269/4260) and 3.87% (165/4260) from Japan and China, respectively. Notably, the JPO had the highest proportion of domestic applicants, closely followed by the CNIPA. Japanese patentees, who also held the second-largest share of granted patents in China and the United States, actively expanded their digital health patents globally. Chinese patentees, despite ranking third based on the nationality of patent applicants in JPO and USPTO, exhibited a notable gap in percentage compared to their American and Japanese counterparts.

### Institution Distribution

A total of 671 institutions or individuals were identified as publishers of patents related to digital health. As detailed in Table 2, Koninklijke Philips emerged as the leading enterprise with 329 published patents, followed by Siemens Healthcare GmbH (252 publications), Shanghai United Imaging Healthcare (151 publications), Samsung Electronics (121 publications), and Canon Medical Systems (118 publications). Notably, all these published patents were granted, with Koninklijke Philips having the highest number of granted patents. The top 10 institution types were exclusively enterprises, with Japanese corporations leading the count at 339 published patents, followed by American companies with 211 published patents, as indicated in Table 2.

**Table 2:** Top 10 institutions with the most published patents (N=15,763).

Institution	Country of origin	Published patents, n (%)
Koninklijke Philips	The Netherlands	^329 (2.09)^
NV Siemens Healthcare GmbH	Germany	252 (1.6)
Shanghai United Imaging Healthcare	China	151 (0.96)
Samsung Electronics Co Ltd	South Korea	121 (0.77)
Canon Medical Systems Corp	Japan	118 (0.75)
Fujifilm Corp	Japan	115 (0.73)
Hitachi Ltd	Japan	106 (0.67)
International Business	United States	106 (0.67)
General Electric Company	United States	105 (0.67)
Biosense Webster (Israel) Ltd	Israel	97 (0.62)

Table 3 highlights the top 10 highly cited institutions, all of which are enterprises. Auris Health Inc and Masimo Corporation stand out with 849 and 983 citations globally, respectively, underscoring their substantial technological influence in the digital health field. It is noteworthy that Lepu (Beijing) Medical Equipment, founded in 1999, is the only Chinese company listed in Table 3.

**Table 3:** Top 10 highly cited institutes in the world.

Institute	Cited frequency, n	Patents, n	Proportion (cited frequency/patents)	Country
Auris Health Inc	849	19	^44.68^	United States
MASIMO corporation	983	28	35.11	United States
Ethicon Llc	700	24	29.17	United States
BERTEC Corporation	251	10	25.10	United States
VIGNET Incorporated	70	5	14.00	United States
Bardy Diagnostics Inc	60	5	12.00	United States
Lepu (Beijing)	149	13	11.46	China
Medical Hi LLC	69	7	9.86	United States
Amazon Technologies	74	8	9.25	United States
Heartflow Inc	43	6	7.17	United States

**Figure 4:**
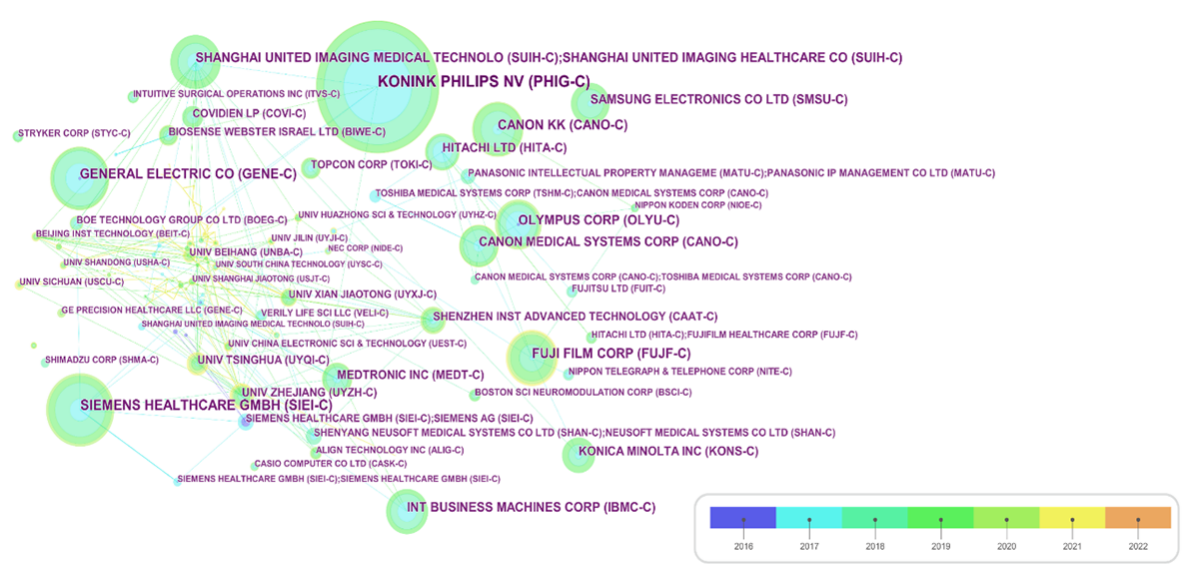
Institutional coauthorship network.

The analysis of coauthorship among institutions revealed noteworthy insights, as depicted in Figure 4. CiteSpace autonomously identified 215 institutions with 212 links, constructing a network illustrating institutional cooperation. The visual representation in Figure 4 incorporates color-coded circles denoting the publication year, node size representing the quantity of published patents, annual-ring width indicating the number of published patents in each year, and link thickness reflecting cooperation strength. Notably, Figure 4 highlights limited international cooperation in the field of digital health among institutions. However, the analysis identifies 2 notable enterprise alliances in Japan. The first alliance involves Fuji Film Corp, whereas the second features Panasonic Intellectual Property Management Corporation collaborating with both Fuji Film and Hitachi. Another noteworthy collaboration in Japan involves Canon, with Olympus maintaining a partnership, albeit with a lower link strength. These collaborative efforts signify strategic partnerships within the Japanese digital health landscape.

Conversely, there were relatively few local corporations collaborating with entities in the United States. General Electric Company exhibited robust research and development capabilities, evident by its limited linkages with other institutions, including universities.

In China, extensive collaboration was observed among hospitals, universities, and research institutions, providing substantial technological support to Shanghai United Imaging Healthcare. This collaborative landscape underscores the active engagement of various entities in China’s digital health sector, fostering advancements through cooperative efforts. Multimedia Appendix 2 illustrates the collaboration direction between Shanghai United Imaging Healthcare, the sole Chinese company listed in Table 3, and Zhongshan Hospital, its primary joint patent application agency, depicted through a word cloud. The primary focus of collaboration between these 2 entities centered around medical imaging, particularly in the fields of tumors and the spine. This collaboration highlights the synergy between Shanghai United Imaging Healthcare, and Zhongshan Hospital in advancing medical imaging technologies, with a specific emphasis on tumor and spine-related applications.

### Inventor Distribution

Table 4 outlines the top 10 most active inventors based on patent filings, featuring 7 Americans and 3 Chinese innovators. This compilation was conducted using Microsoft Excel 2016. Notably, Assaf Govari secured the top position with 43 (0.3%) patents of the total 15763. Frederick E published 31 (0.2%) patents of the total 15,763. Ammar Al Ali, Gary A, Jason L, Yong Wang, and Hairong Zheng collectively published 25 (0.2%) patents of the total 15,763, earning them the third spot in terms of publications. Among the top 10 inventors, 1 hailed from Zhejiang University, the remaining 9 were all affiliated with enterprises, highlighting the significant contributions of both academic and corporate entities in the realm of digital health innovation.

**Table 4:** Top 10 inventors with published patents.

The first inventor^a^	Nationality	Institution	Published patents, n
Assaf	Israel	Biosense Webster (Israel) Ltd	43
Frederick E	United States	Ethicon Llc	31
Ammar Al	United States	Masimo Corporation	25
Gary A	United States	Zoll Medical Corporation	25
Jason L	United States	Ethicon Llc	25
Yong Wang	China	Chison Medical Technologies Co Ltd	25
Hairong Zheng	China	Shenzhen National Res Institute of High-Performance Medical Devices Co Ltd	25
Gust H	United States	Bardy Diagnostics Inc	24
Avi	United States	Align Technology Inc	23
Tao Liu	China	Zhejiang University	20

^a^Inventors publishing the same number of patents were sorted alphabetically.

**Figure 5:**
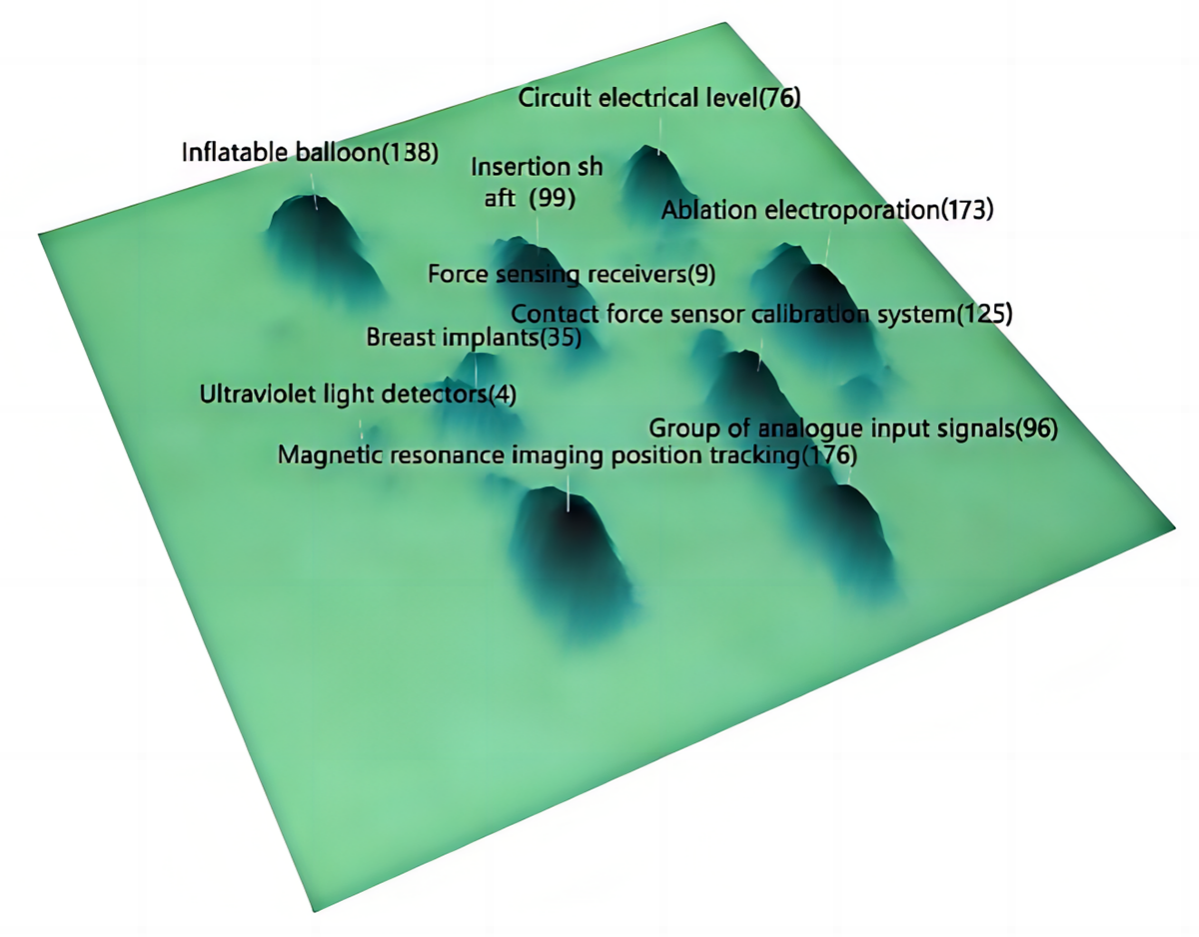
Top 10 technology topics of Assaf Govari.

Assaf Govari, a prominent inventor, served as a fellow in research and development at Biosense Webster, a leading international company specializing in diagnosing and treating heart rhythm disorders [27]. A comprehensive search in the incoPat Patent Data System revealed 565 patent applications listing Assaf Govari as one of the inventors. Figure 5 depicts the analysis of technology themes using the patent 3D sandbox. This innovative visualization represents the competitive landscape of technologies in a 3D topographic map, where peaks signify technology-intensive areas and troughs represent technology gaps. Each dot represents a patent, and proximity indicates relevance. The most frequently mentioned technology theme was data point location tracking for analyzing magnetic resonance imaging data (176 patents). The second most frequent theme was electroporation technology used in ablation procedures during cardiac surgery (173 patents). The third most frequent theme involved an inflatable balloon (138 patents). These insights provide a glimpse into the diverse and impactful contributions of Assaf Govari in advancing technological frontiers in the field of digital health.

Ammar Al Ali held a position as a fellow at Masimo Corporation, a global health care technology enterprise renowned for its research and development of cutting-edge noninvasive patient monitoring techniques. Masimo Corporation also secured the second position among the top 10 leading institutes globally, as indicated in Table 3. Frederick E and Jason L Harris were affiliated with Ethicon Llc, ranking third among the top 10 institutions. Ethicon Llc is widely recognized for its research and development in advanced tissue management. Gust H Bardy served as the founder and chief medical officer of Bardy Diagnostics, a company that held the sixth position among the top 10 institutes globally. Bardy Diagnostics specialized in the development of heart monitors and arrhythmia detection devices, emphasizing the delivery of diagnostically accurate and patient-friendly heart patches and other monitoring solutions. Gust H Bardy oversaw all clinical services related to ambulatory cardiac monitoring within the company.

**Figure 6:**
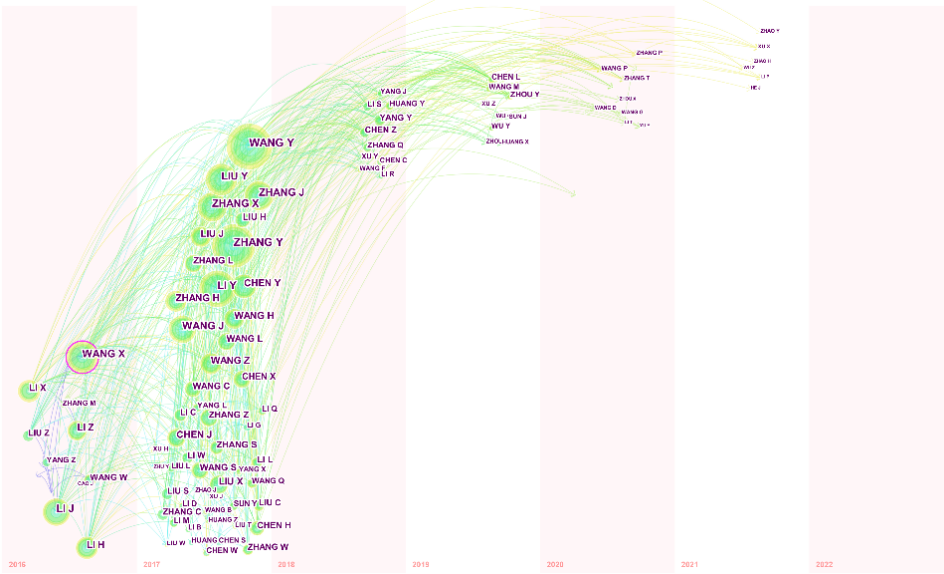
Inventor coauthorship network.

Our analysis extended to coauthorship, using CiteSpace to scrutinize inventor networks. The software meticulously tallied all inventors in each patent, resulting in a coauthorship network of 168 inventors, 639 linkages, and a density of 0.05. Figure 6 visually represents this coauthorship network, with colors of circles denoting the publication year, node size indicating the quantity of published patents, annual-ring width reflecting the number of published patents in each year, and link thickness conveying cooperation strength. Figure 6 reveals that most inventors published patents in 2016 or 2017, and a decline is observed in the number of inventors contributing to technology innovation over subsequent years. This temporal trend offers insights into the dynamics of inventor collaboration, emphasizing the early surge in collaborative efforts in the field of digital health innovation.

Centrality serves as an indicator of a node’s essentiality within a system. CiteSpace uses this metric to discern the relevance of authors or institutions, highlighting those with a centrality of at least 0.1 using a purple circle. In our analysis, Wang X emerged as a highlighted node, and we identified 142 published patents listing Wang X as the inventor.

### Technological Topic and Patents With a High Citation Number

We conducted an investigation into the technological themes of digital health patents using the IPC Code and the DMC. Our searches and categorizations of patent materials were guided by hierarchical classification systems. The IPC, as the sole global classification system for patent documents, serves as a valuable tool for systematically organizing patents, establishing a foundation for the selective release of information, and providing a starting point for research into the state of the art in specific technological fields. The seventh edition of the IPC comprises 8 parts, divided into 120 classes, 628 subclasses, and approximately 69,000 groups [28]. Data for these research categories were extracted from search results in the Derwent Innovation Index database system and the incoPat system.

Table 5 delineates the foremost 10 technology domains within the realm of digital health, as categorized by DMC and IPC numbers. It is noteworthy that a single patent may fall under multiple IPC and DMC classifications, thereby impacting the overall count of patents. Of particular significance in the classification system of DMC were “diagnostic devices” (9081/15,763, 57.61%) and “claimed software products” (3781/15,763, 23.99%), which emerged as the 2 paramount technology subjects in the landscape of digital health patents. In addition, “medical equipment and information systems” (14,579/15,763, 92.49%) played a pivotal role in the overall categorization. Within the spectrum of coding, T01-J (data processing system), P31-A (diagnosis or surgery apparatus), and S05-D (electrical diagnosis) predominated as the primary codes for the sample patents. Notably, the special code listed was B11-C11, denoting “general computing methods and apparatus,” a prevalent technique within the realm of computer science. This underlines its ubiquity and relevance in the digital health patent landscape.

**Table 5:** Top 10 technology topics classified by Derwent Manual Code and International Patent Classification.

Content				Published patents, n
**Derwent Manual Code**				
	T01-J06A	Medical equipment and information systems	14,579	
	P31-A05	Diagnostic devices	9081	
	T01-S03	Claimed software products	3781	
	T01-J10B2	Image analysis	2573	
	S05-D	Electrical diagnosis	1982	
	B11-C11	General computing methods and apparatus	1278	
	S05-D01	Measuring and recording systems	804	
	T01-J10B1	Image enhancement	1028	
	P34-A02	Syringes for removing and introducing fluids into the body	933	
	P31-A01	Surgical tools and instruments	894	
	S05-D07	Diagnostic displays and monitors	756	
**International Patent Classification** **number**				
	A61B5	Measurement for diagnostic purposes; human identification instruments used for radiation	5962	
	A61B6	Diagnosis combined with radiotherapy equipment	1769	
	G06T7	Image analysis	1523	
	G06K9	Method or device for pattern recognition	1214	
	A61B34	Manipulators or robots specially adapted to surgery; computer-assisted surgery	957	
	A61B8	Diagnosis with an ultrasonic, acoustic, or infrasonic waves input device used to convert the data to be processed into a form that can be processed by the computer	878	
	G06F3	Output device used to transfer data from the processor to the output device, for example, interface device equipment for testing eyes	696	
	^A61B3^	Instrument for checking eyes electrotherapy	690	
	A61N1	ICT^a^ dedicated to arranging or managing health care resources or facilities	659	
	G16H40	ICT dedicated to operating medical equipment or devices	650	

^a^ICT: information and communication technology.

From the perspective of the IPC number, pivotal metrics in the field of digital health were evident, with diagnostic and identification measurements (5962/15,763, 37.82%), instruments used for radiation diagnosis (1769/15,763, 11.22%), and image analysis (1532/15,763, 9.72%) emerging as the top 3 technology categories. Within the IPC classification, the sample patents displayed extensive distribution, notably within A61B (diagnosis, surgery, and identification) and G06 (computing and calculating or counting). A noteworthy inclusion in the coding spectrum was the special code G16H04, indicating that information and communication technology has been purposefully designed for the management or administration of health care facilities or resources or the operation of medical equipment and devices. This specialized code underscores the intentional convergence of technology and health care management within the digital health patent landscape.

CiteSpace served as the analytic tool to scrutinize category phrase bursts, facilitating a comprehensive exploration of temporal trends and hot spots within technological themes. Subject categories exhibiting robust burst strengths are indicative of heightened attention from the scientific community during specific periods. These subject categories, derived directly from the DMC of cited patents, encapsulate the primary areas of focus and exploration within a given field during a distinct timeframe. To map the landscape of subject categories, a cocitation network was constructed using the top 20 category themes identified each year. The extraction of the 20 most bursty category themes relied on CiteSpace’s burstiness findings, cataloged in Table 6. The “Year” column denotes the year when a code first appeared, whereas “Strength” quantifies the citation burst intensity for the listed codes. The sorting of these categories is based on the inception year, indicating when a category first exhibited bursts, and is detailed in the fourth column. The “End” column signifies the concluding year of a bursting category, providing a temporal context to the identified trends.

**Table 6:** Top 10 patents according to number of citations.

Publication number	Institute	Year	Cited frequency, n	Client	Client-related product
US10420551B2	Covidien Lp	2019	80	Physicians	Maryland elbow closed cutting surgical instrument
US10980535B2	Ethicon Llc	2021	77	Physicians	Ottava laparoscopic surgery robot
US10464209B2	Auris Health Inc	2019	63	Physicians	Auris robotic endoscopy system
US10539478B2	Auris Health Inc	2020	54	Physicians	Monarch robot endoscope platform
US10416264B2	Hyperfine Research Inc	2019	54	Physicians	Swoop portable MRI^a^
US10426559B2	Auris Health Inc	2019	52	Physicians	Monarch robot endoscope platform
US10140544B1	12 Sigma Technologies	2018	50	Physicians	σ-Discover or Stroke CT^b^
US10070799B2	Pison Technology Inc	2018	49	Patients	Pison AI^c^ neural insights
US10932729B2	Masimo Corporation	2021	47	Patients	Root platform
US10282914B1	Bao Tran; Ha Tran	2019	43	Physicians	VR^d^

^a^MRI: magnetic resonance imaging.

^b^CT: computed tomography.

^c^AI: artificial intelligence.

^d^VR: virtual reality.

**Figure 7:**
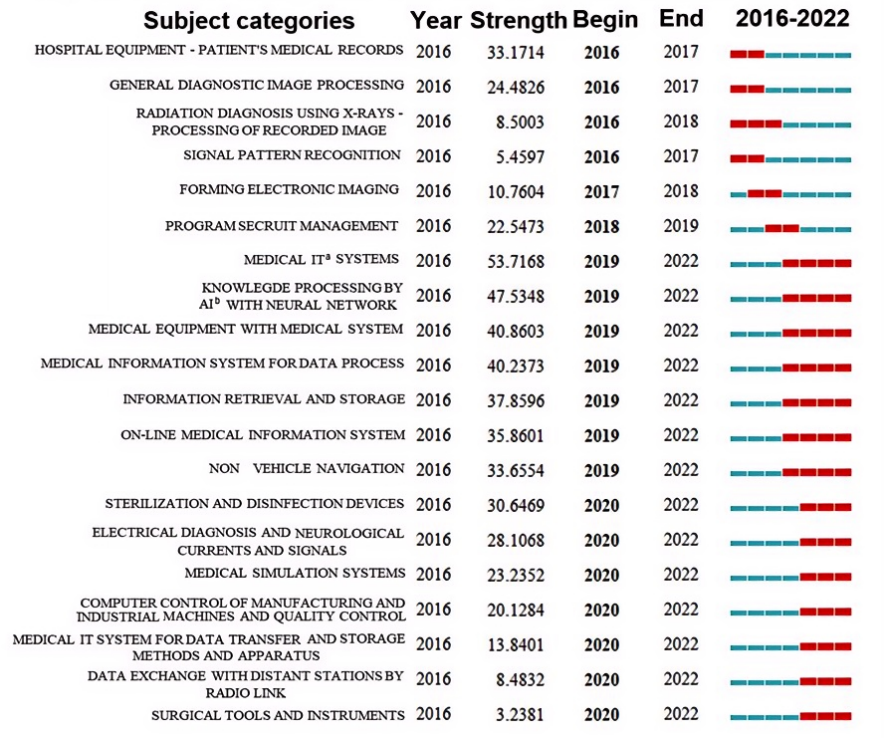
Top 20 subject categories with the strongest citation bursts.

As illustrated in Figure 7, the temporal evolution of digital health technology topics is graphically represented. The red line denotes the commencement year of each topic burst, whereas the subsequent timeline is delineated in green. Figure 7 showcases the top 20 DMC with the most robust burst strength in patents within the digital health domain, characterized by distinct thematic trajectories. The early phase of technological exploration predominantly encompassed topics such as “hospital equipment for patients’ medical records” and “general diagnostic image processing.” These themes garnered widespread citations from 2016 to 2017, exhibiting burst strengths of 33.171 and 24.483, respectively. This implies that patients’ medical records served as a primary data source, and general diagnostic image processing emerged as a principal application scenario for digital health technology during this period.

A subsequent thematic surge in digital health technology research occurred from 2017 to 2019, with a focus on establishing electronic image and data security. The second burst peak materialized in 2019 with the emergence of “medical IT system,” holding the strongest strength in the identified list, and its influence persisted until 2022. In addition, themes such as “knowledge processing by artificial intelligence with neural networks,” “medical equipment with the medical system,” and “medical information system for data processing” coincided with this burst period. Distinct trends unfolded from 2020 to 2022, with themes such as “sterilization and disinfection devices,” “electrical diagnosis for neurological currents and signals,” and “medical simulation systems” gaining prominence. The technology topics with the highest burst strength can be broadly categorized into 4 domains. First, the integration of medical equipment or apparatus with information technology systems, exemplified by themes such as “surgical tools,” “sterilization and disinfection devices,” “medical equipment with the medical system,” and “hospital equipment for patients’ medical records.” Second, themes related to medical information systems, such as “general diagnostic image processing,” “web-based medical information system,” “medical simulation systems,” “medical information technology system for data transfer or storage,” and “medical information system for data processing.” The third category encompasses methods for processing medical data, including themes such as “signal pattern recognition,” “forming electronic image,” “knowledge processing by artificial intelligence with neural networks,” “information retrieval and storage,” and “data exchange with distant stations by radio link.” The last category pertains to the specific application of digital health technology in medical fields, including themes such as “general diagnostic image processing,” “processing of recording image of radiation diagnosis using x-rays,” “electrical diagnosis for neurological currents and signals,” and “nonvehicle navigation.” Furthermore, key technology topics are intricately linked to data security and machine quality, as reflected in the themes of “program security management” and “computerized control of manufacturing of industrial machines and quality control.”

Table 6 outlines the top 10 most cited patents in the digital health field, categorized by the intended users—physicians and patients. Key features of these highly cited patents include a predominant focus on technologies benefiting physicians, with most patentees representing US-based corporations. The granted technologies are primarily method patents, featuring algorithms for tasks such as capturing real-world anatomy, detecting misalignment of robotic arms during surgery, and precise segmentation of medical digital images.

Among the top 10 patents, 4 (40%) prominently center around algorithmic technology. Notably, convolutional neural networks played a pivotal role in advancing the technological landscape. They significantly contributed to the development of image detection in the human brain through magnetic resonance [29] and played a key role in the accurate and efficient segmentation of medical images of human organs [30]. The patent US10426559B2, owned by Auris Health Inc, revealed a method for calibrating a medical instrument equipped with an articulable elongated shaft [31], showcasing advancements in instrument precision. In contrast, the patent titled “Systems and methods for computer-assisted operation” stood out as the sole patent applied by individuals. It unveiled a comprehensive technique for capturing real-world anatomy, implemented in 3D printing within the medical field [32].

Furthermore, 2 additional patents delve into the domain of robotic arms extensively used in surgical procedures. These patents are under the ownership of Auris Health Inc, a Subsidiary Corporation of Ethicon Llc. The patent US10464209B2 presents a system designed to control the position of manipulators both before and during medical procedures. It provides an illustrative example of this technology in action during a diagnostic “and” or “or” therapeutic bronchoscopy procedure [33]. Moreover, the patent addressing the “Detection of misalignment of robotic arms” unveils a system engineered for identifying undesirable forces on manipulators within a surgical machine system. This innovative technique is exemplified in the context of ureteroscopy and laparoscopic procedures [34].

In addition, 2 patents focus on technological advancements aimed at enhancing surgical instruments. The most widely cited patent describes a powered surgical instrument designed to enhance the reliability of communication between the disposable loading unit and the handle assembly. This patent, applied by Covidien Lp, a globally recognized enterprise in the development, production, and sales of health care products, was acquired by Medtronic in 2015 [35]. The patent with the identifier US10980535B2 introduces a surgical instrument tailored for use in endoscopic surgical cutting and fastening procedures [36]. Notably, this patent served to upgrade the Auris Robotic Endoscopy System, the first endoscopic robot developed by Auris Health Inc for the treatment of lung diseases. The system had successfully obtained approval from the US Food and Drug Administration in 2016. It is evident that these 2 highly cited techniques are product patents, conferring a broader scope of protected rights compared to method patents. This distinction underscores the significance of innovations in surgical instrument design and functionality within the realm of digital health.

Moreover, it is noteworthy that the technique titled “Detecting and using body tissue electrical signals,” owned by Pison Technology Inc, holds a prominent position in Table 6 and finds widespread implementation in wearable devices. Pison Technology Inc has articulated the versatile application of this technique across industrial, business, medical, and military domains. Functionally, it is designed for monitoring, control, feedback, actuation, communication, and comprehensive data accumulation and analysis. This innovative technique has practical applications such as monitoring heart rate and electrocardiograph from the wrist or foot. In addition, it is instrumental in measuring or analyzing fitness parameters, including muscular strength and stamina. In the medical field, it plays a crucial role in observing the state and progression of neurological disorders such as amyotrophic lateral sclerosis and other neurodegenerative conditions. The technique’s carrier is a wireless wrist-mounted user interface device, complemented by an app and a universal human-machine interface platform, as detailed in the patent [37]. This comprehensive approach reflects the broader impact and potential of digital health technologies, particularly in the realm of wearable devices and health monitoring.

The final patent to be highlighted is the one titled “Opioid overdose monitoring,” owned by Masimo Corporation. This innovative technique comprises an oximeter designed to be compatible with a handheld monitor for physiological parameters. The system is specifically engineered for monitoring indications of opioid overdose and facilitating the delivery of therapeutic drugs [38].

## Discussion

### Principal Findings

It is crucial to acknowledge that a patent represents an exclusive right granted by a nation to an institution or individual for an invention characterized by innovation, a technical solution, and industrial applicability. Patent information encapsulates a wealth of technological, commercial, and competitor knowledge, with a substantial portion often remaining unpublished. Although bibliometric analyses have been traditionally drawn from articles in digital health–related journals, this study marks the first systematic analysis of digital health technologies using patent documents. This approach serves as a pioneering effort in scientific knowledge dissemination, aiming to identify key nations, inventors, and technological focal points within the realm of digital health. The insights derived from this analysis are intended to provide a valuable reference for researchers and inventors navigating the dynamic landscape of digital health innovation.

In this study, our analysis of patent layouts across different countries revealed Japan as the most active nation in terms of overseas patent distribution, as per statistics from CNIPA, JPO, and USPTO. Notably, we identified distinct cooperation models prevalent in major countries. During the developmental stages of digital health, China, in contrast to the United States and Japan, appeared to be in a startup phase and exhibited a preference for collaborations involving universities, hospitals, and enterprises. In Japan, where digital health industrialization has reached a relatively mature stage, an intercompany cooperation model was found to be more suitable. In the United States, being the most advanced country in digital health industrialization, the need for cooperation seemed less essential, given the presence of formidable companies such as General Electric Company, equipped with robust technological development capabilities.

Furthermore, our examination of patents allowed us to delineate the top 20 subject categories exhibiting the strongest citation bursts. This analysis serves to illuminate the technological development paths within the field of digital health, offering valuable insights into the trajectory of innovation in this domain.

The comparison with the latest bibliometric analysis on digital technologies in health care by Sikandar et al [39], which focused on published articles from 2017 to 2021, reveals interesting disparities in findings. Sikandar et al [39] identified the United Kingdom as the most active country in the research field of digital technology in health care. Noteworthy themes from the sampled articles included “Digital health literacy,” “Digital health for healthcare workers,” “Digital health and covid 19,” and “Applications of digital health” [39]. This comparison underscores a significant divergence in the hot spots and emphases between patents and articles within the digital health domain. While the United Kingdom emerged as the most active country in the article-centric analysis, our study, centered on patents, identified Japan as the most active nation. Furthermore, the themes that gained prominence in articles, such as “Digital health literacy,” were notably absent or had limited mention in the patent landscape. This discrepancy highlights the varied perspectives and priorities of authors and inventors, suggesting that the focus of researchers, as reflected in published articles, may not entirely align with the areas of innovation and patent activity in the digital health field.

The analysis of digital health patents spanning from 2017 to 2021 reveals a notable increase in granted patents in 2018, driven by several key factors. First, supportive policies played a pivotal role in promoting the implementation of digital technology in the medical field. Initiatives such as the “Framework for FDA’s Real World Evidence Program,” “Regulatory decision making for medical devices supported by real-world evidence,” and “Implementation of eHealth Records in Clinical Trial Guidance for Industry,” launched by the US Food and Drug Administration in 2017 and 2018, provided a regulatory framework and impetus for digital health innovation. Furthermore, the Chinese government’s strategic initiatives, as outlined in the “Guidance on Promoting and Regulating the Medical Data Applications” and “Regulations on promoting the development of online healthcare,” released by the General Office of the Chinese State Council in 2016 and 2018, respectively, significantly contributed to the growth of digital health in China. The introduction of the General Data Protection Regulation by the European Union on May 25, 2018, marked a milestone in the formation of industry standards for digital health. This regulation, applicable to any entity handling personal data related to European Union member states, had a global impact on data protection practices. Moreover, the partial coverage of digital health costs by medical insurance in some countries, exemplified by the development of a digital formulary in the United States in 2017, enhanced accessibility and financial viability. This, in turn, stimulated innovation and contributed to the surge in granted patents. In summary, a convergence of regulatory support, strategic government initiatives, the establishment of industry standards, and advancements in insurance coverage collectively fueled the remarkable increase in digital health patents in 2018.

There is no patent layout for digital health in Africa, and possible reasons are as follows. Policy guidance, technological innovation, and financial support are the foundation on which digital health is born and grows. Africa lacks the corresponding economic basis and talent reserve for the development of the digital health. Meanwhile, few corporation giants focusing on digital health are registered in this area because of the turbulent politics situation, poor economic foundation, and low education level, and this area also has no more mature commercial application for the digital health technology.

This study reveals the top countries, key inventors, and hot technologies in the patent landscape for digital therapies, which provides guidance for implementers who are applying for international patents on digital health. Innovation policies for digital health should be formulated and implemented in countries where patents are predominantly applied. The key inventors and hot technologies proposed by the study can be used as both a reference and as an inspiration for inventors to find cooperation partners and pioneer new research and development areas in the near future.

There is no established patent layout for digital health in Africa, and several factors contribute to this absence. The growth of digital health relies on a foundation of policy guidance, technological innovation, and financial support. Unfortunately, Africa lacks the corresponding economic basis and talent reserve necessary for the development of digital health. In addition, the turbulent political situation, poor economic foundation, and low education levels in the region deter major corporations from focusing on digital health, and there is a lack of mature commercial applications for digital health technology. In addition, it is possible that our search methods may impose limitations on the retrieval of patents from the African region.

This study not only underscores the challenges but also reveals key insights into the top countries, key inventors, and emerging technologies in the global patent landscape for digital therapies. These findings offer valuable guidance for international patent applicants in the field of digital health. Countries where patents are predominantly applied should consider formulating and implementing innovation policies for digital health. Moreover, the key inventors and emerging technologies identified in this study can serve as both a reference and an inspiration for inventors. They provide a road map for finding potential cooperation partners and exploring new research and development areas in the near future.

### Limitations

Nevertheless, our study has certain limitations. First, we retrieved only a partial data set from the Derwent Innovations Index database, and some databases were not included. This omission may have resulted in overlooking certain frontier technologies. However, it is worth noting that the Derwent Innovations Index database, from which we collected most published patents, encompasses patents from >50 patent issuing authorities worldwide. Second, our analysis focused solely on granted and valid patents, emphasizing right stability. This approach may overlook some significant patents in the initial application stage. Third, our study was dedicated to analyzing the technical construction of digital health patents through bibliometric analysis. We did not provide a detailed discussion on the content of patents. For a more comprehensive understanding, a systematic patent review in the future is essential. For future work, expanding the scope of patent searches by including searches on Google Scholar could enrich and fill gaps in patents that were not investigated in this study.

### Conclusions

We conducted an analysis of the digital health technology trend from 2017 to 2021 using a data set of 15,763 published patents extracted from the Derwent Innovations Index database. The study not only identified the most productive countries, institutions, and inventors but also delineated the various stages of technology development, key technology categories, and the most cited patents within the digital health domain.

In terms of patent participation rates, China emerged as a frontrunner among the top 10 countries or regions, leading in both the number of patents and technological advancements. This dominance is attributed to China’s concerted efforts in driving digital transformation across various sectors, particularly in medicine. In addition, the substantial patient populations in China contribute significantly to the wealth of data resources available for advancing digital health technologies.

Among the top 10 institutions with the highest number of published patents and the top 10 highly cited institutes globally, all were enterprises. This underscores the pivotal role that corporations play in shaping the patent landscape in the digital health sector. The active engagement of these enterprises is anticipated to yield considerable economic returns in the near future.

Examining the top 10 inventors featured in each patent list, 6 hailed from the United States. Furthermore, the patentees of the leading 10 patents, based on the number of citations, were all Americans. Overall, 90% (9/10) of the highly cited institutes globally also originated from the United States. Undoubtedly, the United States has been a frontrunner in the digital health technology domain. Illustrating a noteworthy collaboration model is Shanghai United Imaging Healthcare, a leader in advanced health care imaging techniques. The company closely collaborated with Zhongshan Hospital, a renowned health institution in the cardiovascular field in China, in the development of digital health solutions.

In our analysis of highly cited patents, we observed that health interventions and improvements in surgical devices were the primary application scenarios for digital health technology. Core to these advancements were algorithms, the focal point of patent-protected technologies. In addition, technologies related to data transfer, storage, and exchange, particularly in the context of telehealth, are anticipated to be hot spots in basic research in the near future.

## Appendix

Multimedia Appendix 1 Nationality distribution of patent applicants within the China National Intellectual Property Administration, Japanese Patent Office, and United States Patent and Trademark Office.

Multimedia Appendix 2 Words cloud of the collaboration between Shanghai United Imaging Healthcare, and Zhongshan Hospital.

## Abbreviations

CNIPA: China National Intellectual Property Administration
DMC: Derwent Manual Code
DTA: Digital Therapeutics Alliance
IPC: International Patent Classification
IPO: intellectual property office
JPO: Japanese patent office
USPTO: United States Patent and Trademark Office

## Data Availability

The data sets generated during and analyzed during this study are available from the corresponding author on reasonable request.
