# Supplementary figures and images for: Evolution of Digital Health and Exploration of Patented Technologies (2017-2021): Bibliometric Analysis
Source: Interact J Med Res. 2024 Jul 11;13:e48259. doi: 10.2196/48259 (PMC11273069; doi:10.2196/48259)

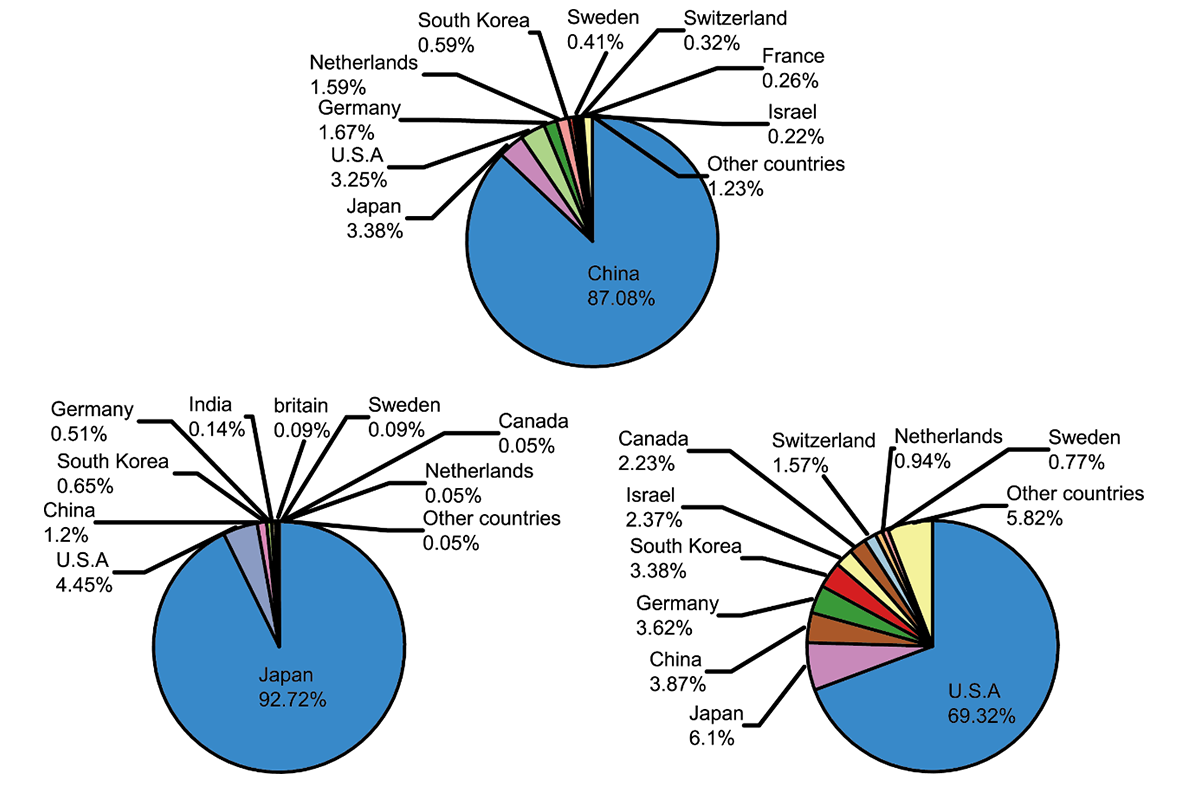

Supplement: Multimedia Appendix 1 [file ijmr_v13i1e48259_app1.png]

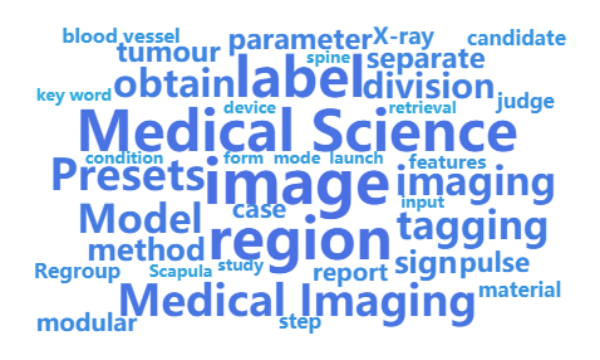

Supplement: Multimedia Appendix 2 [file ijmr_v13i1e48259_app2.png]
